# Fostering Rural High School Students’ Creativity Through Making and Tinkering with 3D Printing

**DOI:** 10.3390/jintelligence14060093

**Published:** 2026-06-01

**Authors:** Yingxiao Qian, Hengtao Tang

**Affiliations:** College of Education, University of South Carolina, Columbia, SC 29208, USA; yingxiao@mailbox.sc.edu

**Keywords:** 3D printing, creativity, making, mixed methods, rural schools

## Abstract

Creativity has been a key driver of innovation; thus, cultivating creative problem solvers is a central goal of STEM education. Making and tinkering practices supported by 3D printing offer a promising avenue for fostering creativity, particularly because the relatively low cost and accessibility of 3D printing make such opportunities feasible in rural educational settings. However, empirical evidence linking these practices to measurable gains in rural students’ creativity remains limited. To address this gap, this study investigated whether integrating making and tinkering experiences through 3D printing affected rural school students’ creativity. A convergent mixed method design was employed in which quantitative and qualitative data were collected in parallel, combining quantitative data from a single-group pretest–posttest design with qualitative insights from semi-structured interviews. After receiving institutional review board approval, parental consent and student assent, this study involved eleven students (ages 16–18) from a rural public high school in the southeastern United States, seven of whom participated in semi-structured interviews. The results indicated a significant increase in students’ creativity scores after participating in the 3D printing session, with a moderate effect size (Cohen’s d = 0.58). Qualitative findings revealed that the hands-on, iterative nature of the 3D printing process fostered students’ creative thinking, in particular, originality and usefulness. The makerspace program provided a tangible platform that allowed students to translate abstract ideas into physical products via rapid prototyping. These findings added preliminary evidence for the potential of integrating 3D printing into educational settings as a means of cultivating creativity, particularly among rural students.

## 1. Introduction

Creativity, the primary source of social and technical innovations (Batey & Furnham, 2006; Mehraein et al., 2023), is defined as an iterative process of generating ideas or artifacts that integrate originality and novelty with practical usefulness (Beghetto & Karwowski, 2017; Runco, 2003). To date, fostering creative problem solvers has become a central goal of science, technology, engineering, and mathematics (STEM) education (Kerr & McKay, 2013; Qian & Choi, 2023, 2025). Cultivating creativity in students enables them to think divergently and thereby come up with original and fluent ideas for them to device innovative solutions (Kaufman et al., 2005; Kettler & Bower, 2017).

Attention has thus shifted toward efforts to cultivate K–12 students’ creativity. While all individuals are endowed with the potential to be creative, their capacity to fulfill their creative potential leads to variations in their creativity (Runco, 2017). Supporting creativity development is particularly important for K–12 students, as educational experiences play a critical role in transforming their creative potential into demonstrated creative ability (Runco, 2003; Runco & Acar, 2012).

However, research has discussed the “creativity gap,” where assessment-driven classroom settings with limited opportunities of inquiry-based, open-ended problem solving may unintentionally thwart students’ creativity development (Runco, 2003, 2017). Moreover, fostering creativity can be particularly challenging in rural contexts due to a lack of extracurricular resources, technological infrastructure, and STEM enrichment programs that foster creativity (Tessman et al., 2025).

The maker movement offers a promising avenue for developing creativity in younger students (Bicer et al., 2017; Eisenberg, 2013; Saorín et al., 2017). Technologies such as 3D printing, which utilize digital fabrication techniques to turn ideas into tangible products, are central to the maker movement (Buechley & Ta, 2023). Moreover, 3D printing allows students to perform making and tinkering practices to generate original design ideas (originality) as well as refine those ideas to formulate effective solutions (usefulness) (Bicer et al., 2017; Saorín et al., 2017). For instance, Saorín et al. (2017) found that collaborative making and tinkering activities via 3D printing allow students to explore diverse solutions to problems, thereby developing and fortifying their divergent thinking skills. Similarly, Bicer et al. (2017) reported that students attending a two-week summer camp focused on 3D computer-aided design, and 3D printing improved their creativity.

Additionally, as the cost of materials and infrastructure decreases, 3D printing technology holds promise for fostering creativity among students from under-resourced areas (Chen et al., 2022; Chen & Cao, 2022; Totuk et al., 2023). Particularly, 3D printing can create meaningful opportunities for rural students to engage in making and tinkering practices that may otherwise be less accessible (Avendano-Uribe et al., 2022; Tang & Qian, 2025a, 2025b). Despite existing evidence highlighting the benefits of integrating 3D printing activities into students’ creativity development, the literature provides limited insight into rural high school students’ learning experiences and how they perceive the development of their creativity during making and tinkering practices. To address this gap, this study tapped into rural students’ experience of making and tinkering with 3D printing and explored how this experience contributed to their perceived growth in creativity. Specifically, two research questions (RQ) were answered below.

RQ1: How does making and tinkering practices in a 3D printing program impact students’ creativity?

RQ2: How do students describe their experience of creativity development in a 3D printing program?

## 2. Literature Review

### 2.1. Creativity and Creative Potential

Definitions of creativity indicate that both originality and effectiveness are the most essential elements (Runco & Jaeger, 2012). Originality is a crucial component of creativity, but it alone does not define creativity (Glăveanu & Beghetto, 2021). Effectiveness is also an indispensable element of creativity, as a truly creative idea or product must be not only innovative but also feasible (Runco & Jaeger, 2012; Sternberg & Lubart, 1999). In short, expressing one’s creativity is to propose novel and fluent ideas in response to a given problem or situation.

Runco (2003) defines creativity as one’s personal pursuit for originality wherein individuals undergo personal interpretation and self-actualization to construct new meanings. This perspective of creativity aligns with some seminal theories that elucidate humans’ cognitive development, such as Piaget’s (1973) “to understand is to invent”. Piaget (1973) posits that one’s understanding is a distinct form of their creativity because, without the construction of new meaning, individuals are limited to rote memorization rather than genuine comprehension. Furthermore, assimilation is viewed as the pathway to individual creativity, while accommodation pertains to convergent insights (Piaget, 1973). Extending Piaget’s theory, Runco (2003) argues that original interpretations are also assimilatory, involving the process of individually absorbing information and then transforming it into original and effective ideas. Building on this view, everyone, including young children, has an inherent tendency to assimilate personal experiences and construct original interpretations, which is referred to as creative potential (Mackinnon, 1965; Runco, 2004; Torrance, 1963). Runco (2003) assumes that all children possess creative potential but notes that preschool children often outperform older children in this regard. Torrance (1968) identifies a “4th grade slump,” which signifies a decline in creative thinking around the fourth grade. This suggests that creative potential may be prominent in early childhood but tends to diminish over time (Runco, 2017, p. xxxiv). Consequently, developing students’ creativity primarily focuses on cultivating their creative potential.

### 2.2. Creativity and Intelligence

Although the importance of creativity has been commonly acknowledged, many teachers lack the necessary expertise to incorporate it into their instruction. To foster student creativity, it is essential to establish a “creative climate” within the classroom (Fasko, 2001). Runco (2017) argues that traditional classrooms, which often resemble a test-like environment, constrain the development of student creativity.

A common misconception is that creativity is equal to intelligence. Traditionally, creative children are solely considered for their high intelligence and giftedness, but defining creativity by intelligence alone is limiting and can be biased because one’s level of intelligence can hinge upon multiple factors (Kim, 2008; Sternberg & Lubart, 1999). Relying on a single-dimensional criterion for giftedness may overlook the exceptional abilities of many gifted underachievers, whose talents are masked by their low performance on standardized tests (Kim, 2008; Runco & Jaeger, 2012). In addition, intelligence is not an innate ability that automatically leads to exceptional performance. Renzulli (1988) proposes that giftedness is a developmental trait requiring continuous cultivation to realize its full potential for creativity and achievement. For instance, the three-ring conception of giftedness examines the intersection of one’s ability, task commitment, and creativity (Renzulli, 2012, 2016; Renzulli & Reis, 2018). Particularly, creativity is regarded as the key that allows students to convert their innate talents into fully developed abilities (Khatena, 1983; Paik et al., 2021). Recent perspectives on giftedness and intelligence have increasingly emphasized their connection with creativity, focusing on students’ creative output.

Conversely, creating a more game-like, open-ended learning environment can better stimulate students’ motivation to develop their creative skills. Additionally, time constraints pose a significant barrier, as students require sufficient time to engage fully in the creative process (Runco, 2017). Addressing these challenges involves overcoming these constraints to create an environment that is conducive to student creativity.

### 2.3. 3D Printing and Creativity

Making and tinkering have been acknowledged for their benefits of serving as drivers of fostering students’ creativity. Saorín et al. (2017) suggest that making and tinkering practices can enhance students’ divergent thinking. 3D printing relies on digital fabrication and rapid prototyping technologies to engage students in making and tinkering practices. As 3D printing becomes more affordable, it holds great promise for integrating design practices into STEM education (Nemorin, 2017). Notably, Hakak et al. (2016) show that students working on 3D interfaces exhibit higher levels of creativity compared to those using 2D interfaces.

The potential of 3D printing technology to develop student creativity lies in its ability to facilitate the generation of novel and original ideas and rapidly verify their effectiveness. First, 3D printing provides an open-ended design environment for students to engage in an iterative process of problem solving, in which they formulate the problem, generate the idea, and continuously evaluate whether they have taken all the possibilities into consideration. Such practices allow students to propose unlimited potential solutions and boost the number of ideas generated (Bevan et al., 2015). Second, this generative environment is particularly conducive to inspiring original ideas, especially when students are given ample time for creative thought (Brod, 2021). 3D printing simplifies the process of transforming abstract concepts into tangible products and enables students to receive rapid feedback on their designs, allowing for quicker iterations and refinements compared to traditional methods. Third, 3D printing can help younger students develop cognitive flexibility, defined as the ability to adapt one’s thinking in response to new and unexpected situations (Canas et al., 2006), by simulating authentic experiences. This setting encourages students to think beyond conventional boundaries and helps cultivate their adaptable mindset of responding creatively to unexpected situations.

## 3. Methodology

This study followed a convergent mixed method design (Creswell & Clark, 2017) that integrated insights from quantitative and qualitative data to answer the research questions. Quantitative research applied surveys to determine if significant changes existed in students’ creativity before and after attending the 3D printing learning experience. Qualitative inquiry focused on students’ descriptions of their learning experience. The findings from the two sources of data were synthesized.

### 3.1. Participants and Contexts

This study took place in a Makerspace program at a rural public high school in the southeastern United States. The program was designed to introduce junior and senior high school students to hands-on making and tinkering activities using 3D printing, with the goal of sparking their interest in STEM fields and future career paths. Participation in the program was open to students without additional inclusion or exclusion criteria. All students received a school announcement about the offering of this program, and those interested could apply to attend it.

The session was offered each semester, and the length of each session varied depending on the school’s schedule. For the program offered in this study, students participated in weekly 45-min sessions across eight weeks. Students only enrolled in this session once and then moved to courses at an advanced level. To support their learning, a teacher from the school with extensive knowledge and experience in 3D printing was assigned to serve as both instructor and facilitator.

Prior to recruiting participants, this study received approval from the Institutional Review Board (IRB) of the authors’ institution. Given that the participants were minors, parental consent and student assent were obtained before the study started. Participation in the research component was voluntary and separate from attending the learning sessions. Students were informed that they could withdraw from the study at any time without penalty or impact on their participation in the makerspace sessions. The teacher/facilitator facilitated the recruitment of participants by disseminating the research description and collecting consent/assent forms.

Fourteen students initially enrolled, but only eleven of them submitted consent and assent forms and agreed to participate in the study voluntarily. Those students who did not return the consent or assent forms continued to attend the sessions, but their data were excluded from this research. All the participants, including six female and five male students, were between 16 and 18 years old. Among them, four identified as African American, four as White/Caucasian, two American Indian or Alaska Native and one as Native Hawaiian or Pacific Islander. The majority of the participants (*n* = 8) were senior students, but none of them had worked on 3D printing projects.

### 3.2. 3D Printing Program

**Figure 1:**
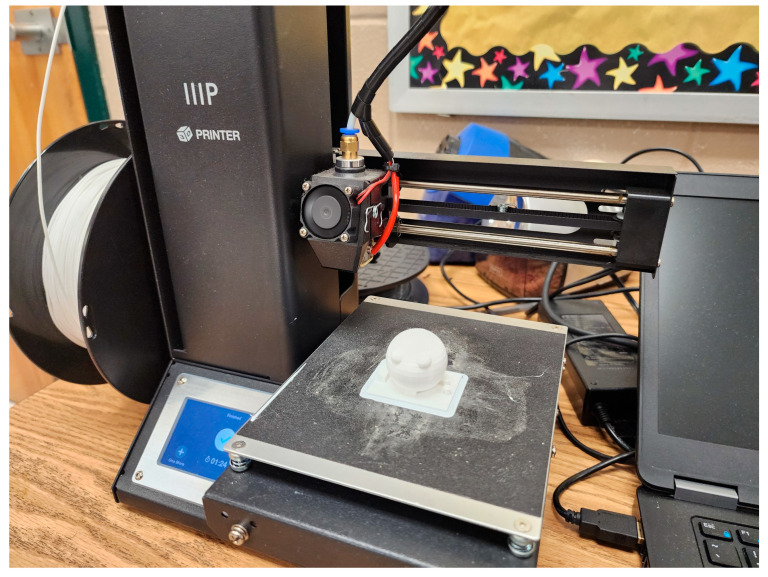
3D printers used for this study.

Participants engaged in open-ended making and tinkering activities, where they built models on their laptops and then produced their creations with 3D printers. The Makerspace session provided one MonoPrice Select Mini 3D Printer (see Figure 1) with a student-to-printer ratio of 14:1. The task included in this study was to design and prototype an original tool for their future lives. This open-ended and personally relevant task was designed to stimulate participants’ creativity and encourage them to develop solutions applicable to their own lives while assessing the novelty and practicality of their ideas.

The teacher/facilitator provided an orientation session covering basic concepts of additive manufacturing, along with the use of 3D printers and related software tools. Then, the teacher/facilitator introduced the task and offered a few examples from fiction videos to stimulate participants’ divergent thinking. Following the orientation, the teacher’s role shifted to that of a facilitator who offered guidance or technical support when participants encountered challenges with designing models or operating the 3D printers.

The task was completed only during the sessions across the period of this study. Participants used AutoCAD Version 25.0 and TinkerCAD to design 3D models, which they then printed using the MonoPrice Select Mini 3D Printer (Monoprice, Inc., Brea, CA, USA). Throughout the sessions, participants worked individually on their laptops, with the facilitator present in the classroom to offer ongoing support. Those who completed their design tasks ahead of schedule had the opportunity to iterate on their designs and refine the digital model for the final printed product. If questions arose during printing, participants were encouraged to revisit their models and revise their designs before trying to print again. This iterative process aimed to give students multiple opportunities to come up with original and effective ideas while exposing them to the importance of testing, evaluation, and continuous improvement in additive manufacturing.

### 3.3. Data Collection

For the quantitative data collection, surveys were used to assess the participants’ creativity in the context of 3D design and printing. Survey items were adapted by the authors from the Personal Creativity Index (McKlin et al., 2018) because this instrument specifically was designed to measure creative expression in artifact creation within STEM learning environments. In the end, a total of ten five-point Likert-type scale items were included with adaptations made to align with the 3D printing makerspace context. Two high school teachers teaching in the same rural school district and two educational measurement faculty read and confirmed the content validity for assessing rural high school students’ creativity. Participants chose responses from 1 (Strongly Disagree) to 5 (Strongly Agree) to indicate the extent to which the statement described their condition. Sample items included “I can come up with new ways to do things in 3D printing” and “I am capable of exploring many different ideas, options, or outcomes in 3D printing”. Survey scores were calculated by averaging responses across the ten items to generate an overall creativity score for each participant. No missing data was recorded. To evaluate the reliability of the survey, Cronbach’s alpha coefficient was calculated. The result confirmed that both pre-test (α = 0.82) and post-test (α = 0.95) showed a high level of internal consistency.

For the qualitative data collection, semi-structured interviews were conducted with participants who were purposively selected based on specific inclusion criteria, including frequency of session attendance, level of engagement, and quality of completed projects based on the notes written by the teacher/facilitator. Particularly, frequency of session attendance was operationalized as the number of sessions each student attended during the period of this study. Level of engagement was determined by indicators such as participants’ active effort in design tasks, iterations of design and revisions, and their intention to explore various design options. In addition, project quality was evaluated by whether students successfully produced a functional object that demonstrated evidence of originality and usefulness. To ensure a diverse range of perspectives, seven participants were chosen in accordance with the maximum variation criterion. The interviews were guided by a semi-structured protocol designed to explore participants’ experiences with the 3D printing intervention and to address any emerging questions (Gikas & Grant, 2013). Sample interview questions included “How do you describe your overall experience of working on 3d printing projects?” and “Have you considered any other options besides the one that you choose?” Each interview was conducted individually in the classroom upon the completion of this study. The interview lasted between 25 and 40 min. For data analysis, each interview was recorded and transcribed with each participant’s permission.

To protect participant privacy, all data (e.g., survey responses, interview transcripts) were de-identified and then stored on password-protected cloud folders only accessible to the research team. Participants’ identification information was removed from transcripts and replaced by assigned participant numbers (e.g., Participant 1, 2).

### 3.4. Data Analysis

For quantitative data, descriptive statistics were provided to summarize the data. To assess the assumption of normality, a Shapiro–Wilk test was conducted, yielding a result of *p* = 0.93, which indicated that the data followed a normal distribution. Consequently, a paired samples *t*-test was used to determine whether the change, if any, in participants’ creativity was significant.

For qualitative data, inductive analysis (Creswell & Creswell, 2017) was conducted by the first author with prior formal training and experience in qualitative research methods and educational research. The coding process included two cycles (Saldaña, 2021). The first cycle included two rounds of coding, in vivo coding and process coding. In vivo coding involved the use of the exact words or phrases from the participants to capture significant statements about their experiences (Saldaña, 2021), such as “trying different designs” and “wanted to create”. Process coding sought to identify actions or processes described by participants (Saldaña, 2021). For this round, the researchers used “-ing” words, such as “creating”, “iterating”, “modeling”, and “fabricating” to assign codes that described behaviors, tasks, or strategies involved in the process of creative problem solving. Then, the codes generated from the two rounds of coding were reviewed by the research team and refined or merged to represent the data. The second cycle included two rounds of pattern coding (Saldaña, 2021). For the first round of pattern coding, all the first-cycle codes were grouped by the proximity in their meaning to formulate patterns, for example, to encompass codes related to participants’ descriptions of how they initiated and applied imagination in their modeling and 3D printing activities. During the second round, these patterns were further analyzed and organized into categories. A total of eight categories were developed, such as “adapting iterative design” and formulating original design”. For the next step, the researcher visualized the relationships among categories to derive overarching themes that captured the participants’ experiences in the 3D printing session. Initially, four themes were proposed; however, the research team met and refined the themes after a peer debriefing session (Tang et al., 2020, 2021). Specifically, the themes “3D printing afforded rapid prototyping for creative problem-solving” and “3D Printing developed student creativity via proposing solutions to real-world problems” were combined to form a more comprehensive theme: “3D printing served a catalyst for creative expression.” Accordingly, the number of categories was reduced to four, with two categories subsuming each theme. Upon mutual agreement, two themes emerged from the data analysis. Then, a summary of the two themes was sent to the participants to ensure they represented their perception of their experience with the 3D printing experience.

To assure the rigor of the findings, rich, direct quotes from the participants’ interviews were also provided (Gikas & Grant, 2013). In addition, participant numbers were also used in the interpretation of the themes below to protect participant privacy.

## 4. Results

### 4.1. Quantitative Findings

Descriptive statistics show that rural students’ creativity increased from pre-test (M = 2.33, SD = 0.59) to post-test (M = 2.94, SD = 0.96). Paired samples *t*-test results confirm that the change in rural students’ creativity was statistically significant, *t*(10) = 3.48, *p* = 0.003, with a moderate effect size (Cohen’s d = 0.58, 95% confidence interval for the mean difference [0.22, 1.00]). Because of the small sample size (*n* = 11), a Wilcoxon signed-rank test was also conducted as a nonparametric sensitivity analysis. The results were consistent with the paired samples *t*-test, Z = 2.45, *p* = 0.02. Overall, the 3D printing intervention significantly improved the students’ creativity.

### 4.2. Qualitative Findings

The qualitative findings uncovered two key themes drawing on the participants’ creative experiences fostered by the 3D printing program, which were further supported by categories, patterns, and specific codes (see Table 1).

#### 4.2.1. Theme 1: 3D Printing Served a Catalyst for Creative Expression

This theme described that 3D printing allowed the participants to express their creativity through participation in hands-on activities and rapidly prototyping design products. Two categories included “formulating original design” and “prototyping solutions to real-world problems”, which supported the two core components of creativity (e.g., originality and usefulness) defined in this study. Participants’ descriptions of exploring multiple design possibilities and infusing imagination illustrated the development of originality, as they were encouraged to experiment with novel designs and translate their imaginative ideas into tangible artifacts. In addition, the emphasis on prototyping solutions to real-world problems and evaluating the effectiveness of those solutions reflects the usefulness dimension of creativity. Overall, the participants generated novel ideas through imagination and considered whether their designs were practically effective. This theme suggested that making and tinkering practices with 3D printing created opportunities for students to integrate imaginative thinking with practical problem solving, reinforcing creativity as the combination of novelty and effectiveness (Beghetto & Karwowski, 2017; Runco, 2003).

The first category, “formulating original design,” highlighted how 3D printing facilitated the participants’ creative exploration and imaginative expression in formulating original designs. This category was characterized by two pattern codes, including “exploring different design possibilities” and “infusing imagination.” The pattern “exploring different design possibilities” recognized the opportunities created by 3D printing, particularly making and tinkering practices, for the participants to experiment with a range of design options. Participant 1 explored various innovative ideas, such as applying 3D printing to biomedical fields and noted how it opened up diverse possibilities in practical contexts. Similarly, Participant 7 shared that 3D printing enabled them to engage in making and tinkering practices and embark on the creation of novel designs or new products.

I considered also working, like, using the 3d printing as something to help with biomedical as in, like creating prosthetics or helping create technologies as well use it as an exterior in the replacement of metal, like metal exterior, rather for a filament exterior.

(Participant 1)

I would say because each one allows you to make a new project, which kind of gives you a more creative way of thinking.

(Participant 7)

The pattern “infusing imagination” reflected a consensus among participants who were captivated by the opportunity to unleash their creativity and apply their imagination in creating novel products. Participant 3 shared a positive experience of exploring various software platforms, which enabled them to design and innovate their own unique ideas. Additionally, Participant 2 emphasized the value of transforming imagination into tangible, lasting creations, which can be cherished indefinitely. This pattern underscored the role of 3D printing as a catalyst for participants to infuse their personal imagination into their designs and foster a deeper engagement with the creative process.

I would say I really enjoyed myself. I was able to explore different software[s], being able to design my own original design and innovate all of that overall, I really do like having my creativity expand.

(Participant 3)

because that’s something you made, like your imagination and your work, something cherished, forever, forever.

(Participant 2)

The second category, “prototyping solutions to real-world problems,” focused on how participants utilized 3D printing to prototype solutions addressing real-world problems, emphasizing two pattern codes such as the “application of creative thinking” and the “effectiveness of creative solutions”. The “application of creative thinking” pattern was evident, as participants used their creativity to prototype solutions built upon to their personal experience. A notable example was Participant 1, who designed fidget toys for children with anxiety or mental health issues. Drawing on a previous middle school project where they had developed a toy for younger children with anxiety, Participant 1 integrated that earlier experience with their creativity to prototype toys that were physically accommodating, socially engaging, age-appropriate, and safe for this target group.

Or making like a making like a fidget toy for children, you know, as a pastime form in case of anxiety or something like along those lines. It was actually um in middle school where we were tasked to make toys and design projects for children with anxiety or mental health issues. So I picked a toy that would accommodate them physically and be social and be age friendly and not all that dangerous for any other younger ages as well.

(Participant 1)

The pattern “effectiveness of creative solutions” illustrated participants’ awareness of testing their prototype or solution for its effectiveness in addition to its originality. Participant 4 invited others to test their model and garnered numerous compliments. Participant 6 aimed to create a unique yet functional shape but ensured that while the design was unusual, it remained intuitive and useful to the user. These experiences demonstrated that 3D printing enabled participants not only to conceptualize creative ideas but also to effectively translate them into practical prototypes that address specific needs.

I gathered participants who would test the model out themselves see how they liked it. It seemed like it gained another a lot of compliments from the respondents. They all seem to enjoy themselves.

(Participant 4)

like unusual when I tried to make it like a unique shape, but also kind of like it’s still like you can understand, like it’s useful.

(Participant 6)

#### 4.2.2. Theme 2: 3D Printing Activities Fostered Students’ Creative Mindset

This theme described that 3D printing cultivated students’ creative mindset geared towards process, iteration, and new perspectives of creativity. Two categories subsumed this theme were “adapting iterative design” and “expanding creative perspectives”, which illustrated engagement with iterative practices of making and tinkering appeared to shape how participants approached the creative process. First, iterative design contributed to the development of creativity by cultivating a process-oriented mindset that facilitated originality and usefulness. While working on 3D models and printing, participants iteratively explored alternative approaches by optimizing their models and testing their design to improve its originality and usefulness. Beyond the novelty of the final project, originality was also reflected in students’ willingness to explore novel approaches throughout the process. Meanwhile, iterative cycles of design, refinement, and optimization led to increasingly functional and effective design, which strengthened the usefulness aspect of creativity. In addition, participants’ reflections on developing new perspectives on creativity indicated making and tinkering practices broadened their understanding of how creative ideas emerged and evolved. Overall, participants’ engagement in iterative cycles of making and tinkering allowed them to develop a more process-oriented mindset of creativity as a balance between imaginative exploration and practical problem solving.

The first category, “adapting iterative design”, illustrated that 3D printing experience cultivated a mindset that valued the design process over the final outcome. This category encompassed two pattern codes: “valuing the process over the outcome” and “learning through iterations.” In the pattern “valuing the process over the outcome,” participants discovered the importance of learning from the design process rather than focusing on design outcomes. Participants described their approach as continuously trying to “find workarounds” and adjusting elements until they achieved a clear understanding of what was wrong, exemplified by resizing objects that were initially too small. Furthermore, Participant 7 emphasized their process of testing “different runs” for a proposed solution to see if they could fix issues or achieve a better outcome. This reflected a shift toward appreciating the iterative nature of design, where the journey of refining and improving holds as much value as the final product.

Try to find workarounds, and if that doesn’t work, I find more workarounds until I get a clear idea of what’s wrong. And whenever I get there, I just try to create, like, the simplest solution, until everything is kind of just fixed. Like with the like I said, with the objects being too small, I would just keep on resizing until everything worked properly. So, it is kind of just fiddling with everything until I figure out what works.

(Participant 6)

I try to think of a solution and I like to test different runs to see if I can fix it or have a better outcome.

(Participant 7)

The pattern “learning through iterations” demonstrated participants’ reflection of learning from iterations of design, troubleshooting, and optimization. Participant 5 highlighted the necessity of identifying main challenges and exploring different solutions efficiently, focusing on learning from each attempt. Participant 4 shared their experience of iteratively adjusting their designs to fix initial sizing errors. Overall, the participants recognized that each iteration contributed to solidifying their model design and 3D printing products. The emphasis on iterative learning fostered a creative mindset that was open to exploration and continuous improvement.

Identify what, first of all, what the main challenges would be, find different ways to how to solve it, and not only quickly, but to make sure it’s efficient and to make it right and also learn from it.

(Participant 5)

Oh god! Yes, I kept on making things that were too small and it would end up with so much of what’s, what’s the word for it, like the foundation, the structure, the scaffolding. It would end up with so much scaffolding that the scaffolding was bigger than the object I made.

(Participant 4)

The second category, “expanding creative perspectives”, noted that 3D printing experience broadened participants’ creative thinking abilities and allowed them to find new ways to interpret creativity. This category included two pattern codes: “developing creative thinking” and “embracing new lenses of creativity.” The pattern “developing creative thinking,” recorded the experience that nurtured the participants’ creativity. Participant 3 felt 3D printing processes acted as “kind of like a check to my creativity” and motivated them to expand ways of thinking to make their design novel and effective. Participant 4 anticipated that engaging with 3D printing would enable them to think more creatively as well as critically.

I think that it will allow me to expand [my] my knowledge of the subject, and be able to have a different way of thinking, more critical thinking, as well as artistic, creative.

(Participant 4)

The pattern “embracing new lenses of creativity” depicted participants’ new lens of perception about creativity in simplicity and unconventional ideas. Participant 2 interpreted their new lens of understanding creativity as “kind of creative with the simplicity.” Additionally, Participant 7 mentioned being creative by using something “that typically wouldn’t be expected,” indicating a shift towards new perspectives of creativity. Overall, the 3D printing experience encouraged participants to see beyond conventional approaches and embrace a mindset that values originality and effectiveness.

I guess it was, I wouldn’t say it was a stroke of genius or anything, but [it was,] it was fun to me to realize that…it was kind of creative with the simplicity.

(Participant 2)

Now I feel, creative in the sense that, like, [you are,] you are using something that typically would not be expected to.

(Participant 7)

## 5. Discussion

The integration of quantitative results and qualitative findings contributed to the existing literature by showing that engaging with 3D printing activities allowed rural students to reinforce the originality of their ideas and also provided them with an opportunity to iteratively refine these ideas and transform them into tangible products. Quantitative results, despite the small sample size of this study, showed that making and tinkering practices driven by 3D printing can play a role in fostering creativity among rural students. The rural students’ increased creativity observed in this study aligned with previous research emphasizing the value of hands-on, open-ended projects, such as engaging students in making and tinkering practices with 3D printing, for fostering their creativity (Avendano-Uribe et al., 2022; Chen et al., 2022; Pinar et al., 2025; Tang et al., 2024; Totuk et al., 2023). Qualitative data further enriched quantitative results by illustrating how those making and tinkering practices impacted rural high school students’ creativity. Participants expressed that 3D printing acted as a powerful catalyst for their creativity and allowed them to transform abstract ideas into tangible objects. This process sharpened their creative thinking and facilitated rapid prototyping of their solutions, which became an essential part of the process for students to come up with their original and effective design (Pinar et al., 2025; Saorín et al., 2017). This aligns with the idea that creativity is not just about generating ideas but also about evaluating and refining them (Runco & Jaeger, 2012). In addition, the iterative cycles of design, testing, and refinement inherent in 3D printing practices mirror the real-world context and foster students’ creative mindset oriented towards iterations and novel perspectives of creativity. These findings are consistent with earlier studies that highlight the value of 3D printing in affording iterative design process, where students are encouraged to experiment, fail, and learn from their mistakes as well as convert abstract ideas into concrete artifacts (Bicer et al., 2017; Saorín et al., 2017; Tang et al., 2023). The open-ended nature of the 3D printing projects allowed students to explore various solutions, leading to an increase in the number and originality of ideas generated.

### 5.1. Practical Implication

This study highlights the importance of providing a learning environment that supports creativity, particularly for rural students. Traditional classroom settings, often characterized by rigid structures and standardized testing, can stifle creativity (Runco, 2017). In contrast, the 3D printing program offered a more flexible, student-centered learning environment that encouraged exploration and experimentation. This supports the argument that fostering creativity requires not only technologies but also effective learning environments that nurture creative expression (Fasko, 2001). The enthusiasm and engagement expressed by the participants in this study suggest that 3D printing can serve as a powerful tool in creating such a climate of creativity.

### 5.2. Limitation and Future Research

This study was limited by a few constraints. First, the relatively small sample size and a lack of control group limited the generalizability of the research findings, but the implications of implementing makerspace to cultivate student creativity can be replicated in other rural settings. Second, the research mainly relied on self-reported measures of student creativity, which may bring in subjectivity (e.g., social desirability or students’ perceptions of expected outcomes) of the participants regarding their creativity. Third, the observed increase in students’ creativity may partly result from a novelty effect, as exposure to 3D printing could enhance students’ creativity in the short term. Similarly, the role of the facilitator and the supportive instructional context may have also contributed to the students’ positive experiences, making it difficult to isolate the effect of the technology itself.

Future research may consider examining the impact of rural students’ makerspace experience on their creativity in a large sample following a longitudinal experimental design. Moreover, future research may include a comparison between rural and urban settings to determine the feasibility of 3D printing interventions for schools with relatively limited resources. In addition, future research may consider multimodal measures or integrate process-based evaluations of student creativity to offset the limitation of self-reported measures.

## 6. Conclusions

Creativity, often defined as the generation of novel and useful ideas, is a cornerstone of innovation in STEM fields (Batey & Furnham, 2006; Mehraein et al., 2023). This study added to the growing body of literature that advocates for the integration of 3D printing to enhance creativity, especially among rural students. The combination of quantitative and qualitative findings provided preliminary evidence of how 3D printing could be leveraged to cultivate creative potential and offered valuable insights on students’ experience and perception of how making and tinkering practices engaged them in creative development. The findings provided practical implications for educators seeking to develop innovative making-based STEM programs.

## Figures and Tables

**Table 1:** Themes, categories, patterns and sample codes.

Themes	Categories	Patterns	Codes
3D printing served as a catalyst for creative expression.	Formulating original design	Exploring different design possibilities	“another approach”; innovating; creating novel solutions
		Infusing imagination	“having creativity expand”; designing with imagination
	Prototyping solutions to real-world problems	Application of creative thinking	“help kids with anxiety”; accommodating
		Effectiveness of creative solutions	“unique…but understand”; testing the model
3D printing activities fostered students’ creative mindset.	Adapting iterative design	Valuing the process over the outcome	“fiddling with everything”; discovering
		Learning through iterations	“tweaking the design”; resizing
	Expanding creative perspectives	Developing creative thinking	“different way of thinking”; checking my creativity
		Embracing a new lens of creativity	“creative with simplicity”; making sense of creativity

## Data Availability

The datasets presented in this article are not readily available because of the IRB requirement.

## Abbreviations

The following abbreviations are used in this manuscript:

STEM	Science, technology, engineering, and mathematics
3D	Three-dimensional
